# Supplementary material for: Different cellular and molecular responses of Bovine milk phagocytes to persistent and transient strains of Streptococcus uberis causing mastitis
Source: PLoS One. 2024 Jan 11;19(1):e0295547. doi: 10.1371/journal.pone.0295547 (PMC10783761; doi:10.1371/journal.pone.0295547)
Supplement: S1 File — (PDF) [file pone.0295547.s001.pdf]

**S1 Table. Data of CMT score and Somatic cell count of the samples used in the study.**

| Sample no. | Quarter | CMT score | SCC (× $10^3$ cells/ml) |
|---|---|---|---|
| 1 | Left rare | 2 | 3924 |
| 2 | Right front | 2 | 1239 |
| 3 | Right rare | 2 | 628 |
| 4 | Right front | 2 | 3151 |
| 5 | Right front | 2 | 7849 |
| 6 | Left front | 2 | 4551 |
| 7 | Left front | 2 | 2521 |
| 8 | Left rare | 2 | 3187 |
| 9 | Left front | 2 | 6597 |
| 10 | Left front | 2 | 1955 |
| 11 | Right front | 2 | 1798 |
| 12 | Right rare | 2 | 5590 |
| 13 | Left front | 2 | 6870 |
| 14 | Left rare | 2 | 857 |
| 15 | Right front | 2 | 9953 |
| 16 | Left front | 2 | 4006 |
| 17 | Left front | 2 | 1587 |
| 18 | Left rare | 2 | 3205 |
| 19 | Right rare | 2 | 6728 |
| 20 | Right front | 2 | 1261 |

**S2 Table. RNA concentration and purity values.**

| Sample no. | RNA concentration | A260/A280 |
|---|---|---|
| 1 | 656.7 | 1.50 |
| 2 | 447.3 | 1.84 |
| 3 | 423.4 | 1.89 |
| 4 | 758.8 | 1.52 |
| 5 | 1039.6 | 1.80 |
| 6 | 655.5 | 1.85 |
| 7 | 746.0 | 1.82 |
| 8 | 633.5 | 1.90 |
| 9 | 1024.8 | 1.92 |
| 10 | 863.4 | 1.50 |
| 11 | 489.0 | 1.92 |
| 12 | 414.7 | 1.85 |
| 13 | 487.9 | 1.95 |
| 14 | 1140.3 | 1.82 |
| 15 | 796.5 | 1.85 |
| 16 | 688.2 | 1.52 |
| 17 | 421.6 | 1.86 |
| 18 | 897.0 | 1.60 |
| 19 | 1004.6 | 1.80 |
| 20 | 887.9 | 1.89 |

**S3 Table. Primer and gene efficiencies.**

| Gene | Log dilution | Ct | Correlation coefficient ($R^2$) | Equation of a standard curve | Slope | Primer Efficiency (E) |
|---|---|---|---|---|---|---|
| *TLR1* | $10^{-1}$ to $10^{-6}$ | 18-34 | 0.9564 | Y = -3.140*X + 38.77 | -3.14 | 108.19 |
| *TLR2* | $10^{-1}$ to $10^{-6}$ | 16-32 | 0.9711 | Y = -3.106*X + 36.16 | -3.106 | 109.87 |
| *TLR6* | $10^{-1}$ to $10^{-6}$ | 17-34 | 0.9933 | Y = -3.372*X + 38.21 | -3.372 | 97.95 |
| *LAMP1* | $10^{-1}$ to $10^{-6}$ | 17-33 | 0.9727 | Y = -3.048*X + 37.35 | -3.048 | 112.86 |
| *RAC1* | $10^{-1}$ to $10^{-6}$ | 16-33 | 0.9713 | Y = -3.608*X + 37.28 | -3.608 | 89.30 |
| *SOD1* | $10^{-1}$ to $10^{-6}$ | 17-33 | 0.9784 | Y = -3.028*X + 36.98 | -3.028 | 113.92 |
| *NOX1* | $10^{-1}$ to $10^{-6}$ | 18-34 | 0.9863 | Y = -3.339*X + 37.81 | -3.339 | 99.29 |
