# Supplementary material for: Different cellular and molecular responses of Bovine milk phagocytes to persistent and transient strains of Streptococcus uberis causing mastitis
Source: PLoS One. 2024 Jan 11;19(1):e0295547. doi: 10.1371/journal.pone.0295547 (PMC10783761; doi:10.1371/journal.pone.0295547)
Supplement: S2 File — (PDF) [file pone.0295547.s002.pdf]

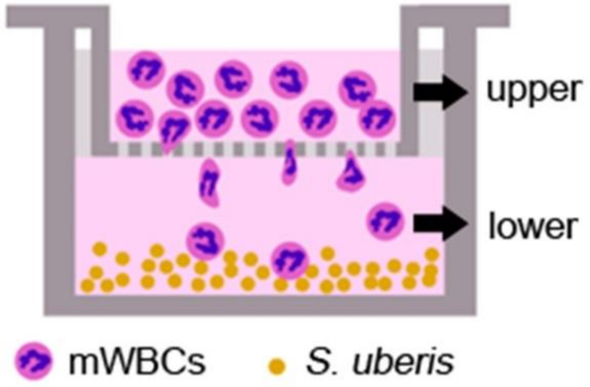

S1 Fig. Illustration of Transwell culture system.

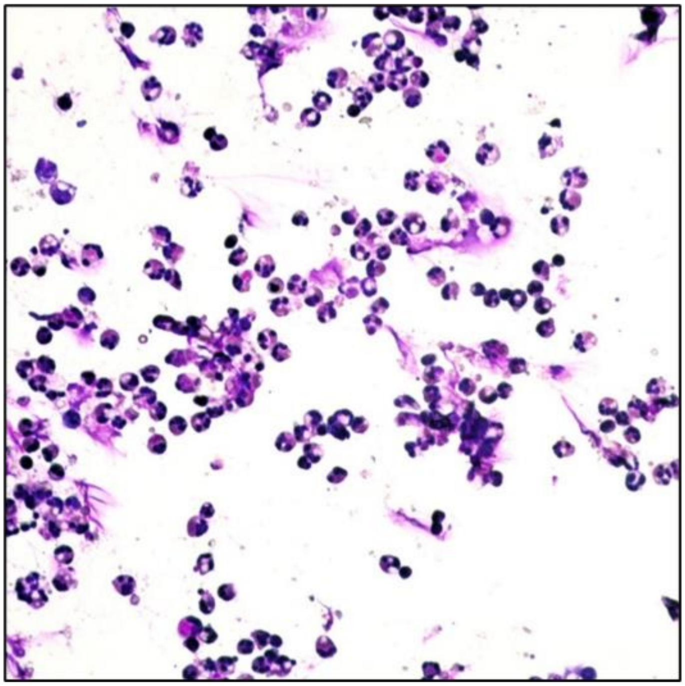

S2 Fig. A cytospin preparation slide was used to check the differential cells of mWBC.

SU03	SU04	
aaaT	aaaT	
aacA-aphD	aacA-aphD	
aadK		Aminoglycoside 6-adenylyltransferase
accA	accA	
accB	accB	
accC	accC	
accD	accD	
acdA	acdA_1	Acyl-CoA dehydrogenase
	acdA_2	
ackA	ackA	
acoA	acoA	
acpP_1	acpP_1	
acpP_2	acpP_2	
acpS	acpS	
act		
	actP	Copper-transporting P-type ATPase
acyP	acyP	
adcR	adcR	
addA	addA	
yvcJ	yvcJ	
yvdD	yvdD	
	yvdT	putative HTH-type transcriptional regula
yvgN	yvgN	
ywlC	ywlC	
ywpJ	ywpJ	
yxdL	yxdL	
yxdM		ABC transporter permease protein Yxd
yxeM	yxeM	
yxeN	yxeN	
yyaP	yyaP	
yybR	yybR	
yycJ	yycJ	
zitB	zitB	
	zntB	Zinc transport protein ZntB
znuA	znuA_1	
	znuA_2	High-affinity zinc uptake system binding
znuB	znuB	
znuC	znuC	
zosA	zosA	
zupT	zupT	

SU03	SU04	
metK	metK	
metN	metN	
metP	metP	
metQ	metQ	
mfd	mfd	
mgsR	mgsR	
mhqA		Putative ring-cleaving dioxygenase MhqA
mhqD		Putative hydrolase MhqD
mhqO		Putative ring-cleaving dioxygenase MhqO
mhqR	mhqR	
miaA	miaA	
miaB	miaB	
mii	mii	
misCA	misCA	
mltF_1	mltF_1	
mltF_2	mltF_2	
mltG	mltG	
	mngB	Mannosylglycerate hydrolase
gtf3_1	gtf3_1	
gtf3_2	gtf3_2	
gtfA	gtfA	
gtfB	gtfB	
guaA	guaA	
guaB	guaB	
guaC	guaC	
gyrA	gyrA	
gyrB	gyrB	
hasB		UDP-glucose 6-dehydrogenase
hbd	hbd_1	
	hbd_2	
hdfR_1	hdfR	
hdfR_2		HTH-type transcriptional regulator HdfR
hemW	hemW	
hepT	hepT	
	hexR	HTH-type transcriptional regulator HexR
hflX	hflX	
hipO	hipO	
hisC	hisC	
hisJ_1	hisJ_1	

S3 Fig. Various bioinformatic tools to analyze shotgun sequences for persistent (SU03) and transient strains (SU04).

```
LOCUS       LLLCNIDG_1         570853 bp    DNA    linear    16-JUL-2022
DEFINITION  Streptococcus uberis strain strain.
ACCESSION
VERSION
KEYWORDS    .
SOURCE      Streptococcus uberis
  ORGANISM  Streptococcus uberis
            Unclassified.
COMMENT     Annotated using prokka 1.14.6 from
            https://github.com/tseemann/prokka.
FEATURES             Location/Qualifiers
     source          1..570853
                     /organism="Streptococcus uberis"
                     /mol_type="genomic DNA"
                     /strain="strain"
     gene            421..1011
                     /gene="clpP"
                     /locus_tag="LLLCNIDG_00001"
     CDS             421..1011
                     /gene="clpP"
                     /locus_tag="LLLCNIDG_00001"
                     /EC_number="3.4.21.92"
                     /inference="ab initio prediction:Prodigal:002006"
                     /inference="similar to AA sequence:UniProtKB:Q9ZAB0"
                     /codon_start=1
                     /transl_table=11
                     /product="ATP-dependent Clp protease proteolytic subunit"
                     /protein_id="Prokka:LLLCNIDG_00001"
                     /db_xref="COG:COG0740"
                     /translation="MIPVVIEQTSRGERSYDIYSRLLKDRIIMLTGPVEDNMANSIIA
```

S4 Fig. An example of a GenBank file for persistent *S. uberis*.

$$Efficiency\ (\%) = \left(10^{\frac{-1}{Slope}} - 1\right) \times 100$$

S5 Fig. The equation for calculation of the efficiency of a primer (%).

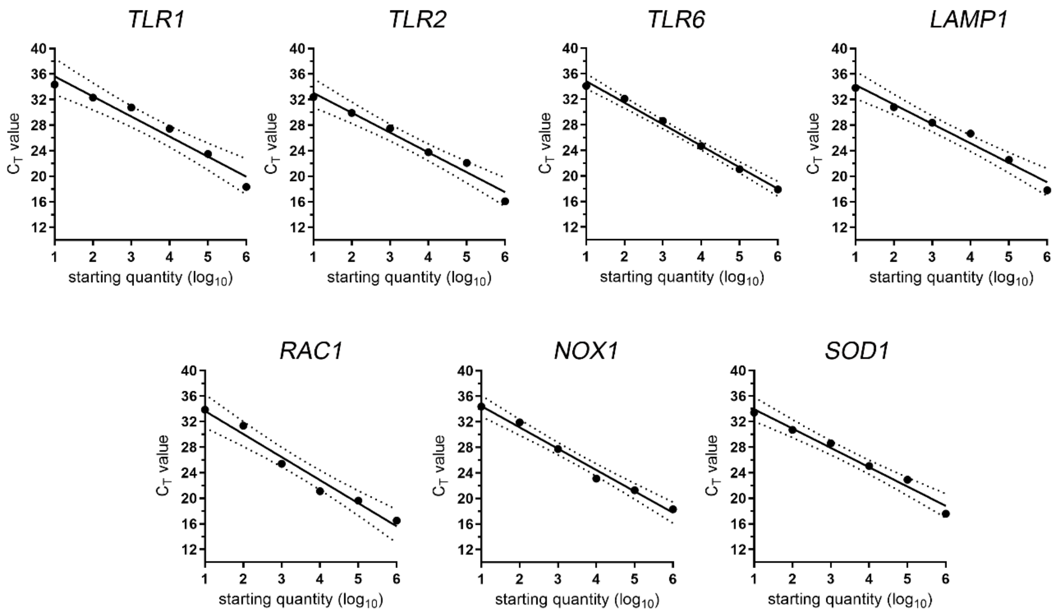

S6 Fig. Results of the efficiencies of primers in this study.

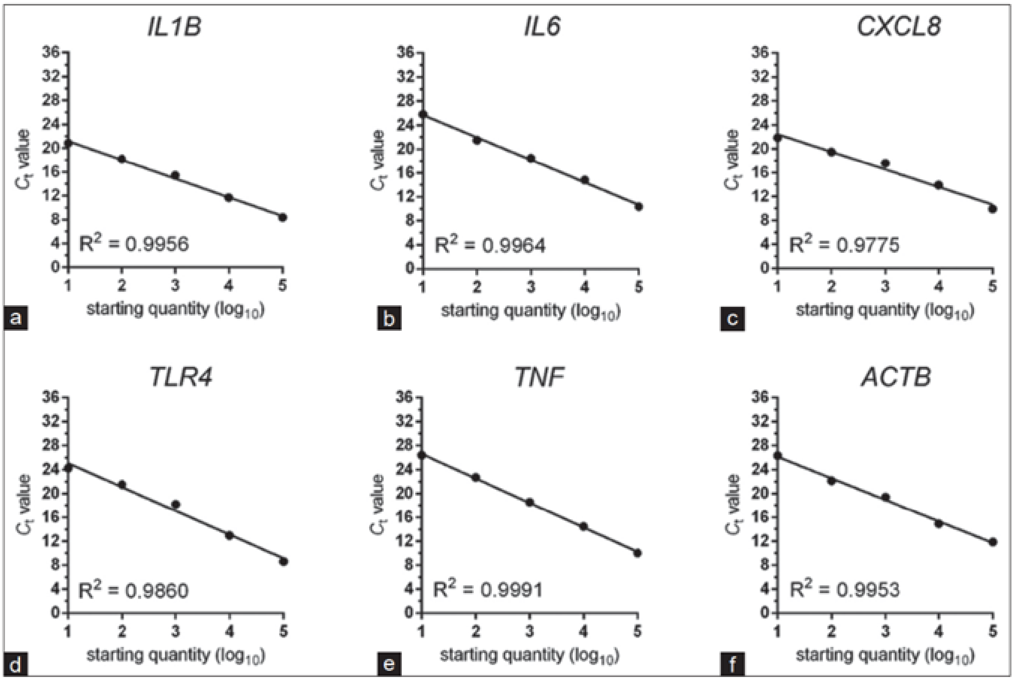

S7 Fig. The primer efficiencies for the genes IL1B, TNF, CXCL8, and ACTB. Adopted the information from : Chuammitri P, Srikok S, Saipinta D, Boonyayatra S. The effects of quercetin on microRNA and inflammatory gene expression in lipopolysaccharide-stimulated bovine neutrophils. Vet World. 2017;10(4):403-10. doi: 10.14202/vetworld.2017.403-410.
