# Supplementary material for: Different cellular and molecular responses of Bovine milk phagocytes to persistent and transient strains of Streptococcus uberis causing mastitis
Source: PLoS One. 2024 Jan 11;19(1):e0295547. doi: 10.1371/journal.pone.0295547 (PMC10783761; doi:10.1371/journal.pone.0295547)
Supplement: Attachment — Submitted filename: Response to reviewer.docx [file pone.0295547.s003.docx]

**Manuscript ID: PONE-D-23-14210**

**Title: Different cellular and molecular responses of bovine milk phagocytes to persistent and transient strains of *Streptococcus uberis* causing mastitis.**

The authors appreciate all comments and suggestions from the reviewers. We have read and understood all of your comments and suggestions, and we have responded to all of your questions below.

Two versions of the article were provided to the reviewers: a clean version (Manuscript) and a version with tracked changes (Revised Manuscript with Track Changes).

**Reviewers' comments:**

**Reviewer 2:**

1. I still do not think the authors have addressed the use of 1 internal control gene. The authors seem to suggest that an acceptable primer efficiency could be used as an indicator that one control gene is sufficient. I don't agree. The authors should report the coefficient of variation for beta actin across all samples or use geNORM as a tool to determine if 1 internal control gene was sufficient. The issue with using one internal control gene is that the internal control gene may not be stable across your samples. This particular issue is not necessarily related with the primer (primer efficiency). Potentially, the treatments (different strains of S. uberis) could impact the expression and mRNA abundance of beta actin, which would distort your ability to assess treatment (different strains of S. uberis) effects on your target genes.

**Response 1:** Thank you for your comments and suggestions. We agreed to calculate the coefficient of variation for the β-actin gene as an internal control in real-time PCR.

To evaluate the coefficient of variation (CV) of β-actin gene as an internal control in real-time PCR, we retrieved our data on the Ct values of the gene in different treatments (unstimulated control, persistent *S. uberis*, and transient *S. uberis*). The triplicate data from the experiment is arranged in the figure below (Figure 1-Left).

The intra-CV of β-actin is the coefficient of variation within each sample/treatment group. It is calculated by dividing the standard deviation (SD) of the Ct values within each triplicate sample/treatment group by the mean Ct value of that triplicate sample/group. The inter-CV cannot be calculated because the treatment groups were not performed on separate plates. The CV of gene expression is a measure of how much variation there is in the expression levels of a gene across different samples/treatments. It is calculated as the ratio of the standard deviation (SD) of the gene expression to the mean expression level, expressed as a percentage. We calculate the intra-CV using the following equation: % CV = (SD / Mean) × 100

**Figure 1** Data on the Ct values of the β-actin gene (Left) and average intra-CV values of β-actin in different treatments (Right).

The intra-CV is a measure of the precision of the assay, which is how reproducible the results are. It is calculated by measuring the variation between three replicates of the same sample (mWBC) within the β-actin gene by real-time PCR. A low intra-CV indicates that the assay is precise and that the results are reproducible. We have calculated the average intra-CV values of 1.588%, 0.977%, and 0.824% for unstimulated, transient, and persistent *S. uberis*, respectively (Figure 1- Right).

The intra-CV and inter-CV should both be below 10% for the beta actin gene to be a reliable reference gene. If the CVs are too high, it means that the gene is not expressed consistently in all the samples, and it cannot be used to normalize the expression of other genes.

**Solution :** To determine that only 1 internal control gene was sufficient, we report the coefficient of variation for beta actin across all samples by adding a sentence in **line 218 - 221** of the revised manuscript: “The averages of intra-coefficient of variation (CV) values of *ACTB* were 1.588%, 0.977%, and 0.824% for unstimulated, stimulated with transient, and persistent *S. uberis*, respectively, which are below 10% indicating that ACTB was a reliable reference gene.”

2. I also think the authors should have measured RNA integrity and reported it in the manuscript, although I wouldn't bother trying to measure it now because the RNA is 4 years old. Please consider this in the future.

**Response 2:** We appreciate your feedback on the measurement of RNA integrity. We agree that it is important to assess RNA integrity, and we will take this into account in our future research.

3. Line 24 - I would say isolated from milk collected from dairy cows with mastitis, or something similar like mastitis milk?

**Response 3:** The authors have checked and corrected the information.

We have edited the text in **line 25** of the revised manuscript from “…isolated from mastitis in dairy cattle.” to " ….isolated from milk collected from dairy cows with mastitis.”

**Reviewer 3:**

4. This reviewer is however confused by results from figure 2 where the authors have used a fluorescently labeled S. uberis strain to assess phagocytosis. I understand that labeling with persistent strain was not possible. Yet, with the transient strain, how do the authors explain that HBSS control group have stronger fluorescence than the cells incubated with the bacteria? If I understand correctly, there should not be any fluorescence with the HBSS group.

**Response 4:** Thank you for your comments. We also agree that the finding could confuse readers, especially the fluorescence in the HBSS group. In our study design, we would like to compare phagocytosis between mWBC stimulated with either transient or persistent S. uberis. Unfortunately, we could not perform the comparison as explained in lines 421 – 427. The finding of the lower phagocytosis activity of mWBCs stimulated with S. uberis than mWBCs stimulated with HBSS might be due to the fluorescent response of mWBC after stimulation. The fluorescence detected in WBC stimulated with HBSS was attributed to autofluorescence with more intensity in the activated PMNs [58] as the mWBC in this study. The less fluorescence mWBC stimulated with transient S. uberis might be caused by blocking the emission of fluorescent light or quenching the fluorescence of S. uberis within mWBCs by absorbing the excitation light, resulting in a weaker fluorescent intensity as depicted in Fig 2B.

To make it clearer, we revised the sentences in lines 419 – 434 of the revised manuscript:

**Delete** “For phagocytosis in mWBCs measured using flow cytometry, fluorescently labeled transient S. uberis-stimulated mWBCs showed significantly less phagocytosis than HBSS-stimulated mWBCs (Fig 2). These results indicate that there was less phagocytosis activity of mWBCs stimulated with S. uberis compared to a negative control. In general, S. uberis has previously been shown to resist phagocytosis in vitro and in vivo [56, 57]. In addition, the lower phagocytosis in response to transient S. uberis might also be related to the use of mWBCs.” In **Lines 400-406**.

**Add** “The phagocytosis measured using flow cytometry in this study was firstly designed to compare the activity between fluorescently labeled transient and persistent S. uberis stimulated mWBCs.” in **Lines 419 – 421** of the revised manuscript.

**Add** “The finding of the lower phagocytosis activity of mWBCs stimulated with S. uberis than mWBCs stimulated with HBSS (Fig 2) might be due to the fluorescent response of mWBC after stimulation. The fluorescence detected in WBC stimulated with HBSS was attributed to autofluorescence with more intensity in the activated PMNs [57] as the mWBC used in this study. The less fluorescence mWBC stimulated with transient S. uberis might be caused by blocking the emission of fluorescent light or quenching the fluorescence of S. uberis within mWBCs by absorbing the excitation light, resulting in a weaker fluorescent intensity as depicted in Fig 2B.” in **Lines 428 – 434** of the revised manuscript.

We look forward to hearing from you regarding our re-submission.

Sincerely,

Corresponding authors and on behalf of the authors
