# Supplementary material for: Different cellular and molecular responses of Bovine milk phagocytes to persistent and transient strains of Streptococcus uberis causing mastitis
Source: PLoS One. 2024 Jan 11;19(1):e0295547. doi: 10.1371/journal.pone.0295547 (PMC10783761; doi:10.1371/journal.pone.0295547)
Supplement: Attachment — Submitted filename: Response to Reviewers.docx [file pone.0295547.s004.docx]

**Manuscript ID: PONE-D-23-14210**

**Title: Different cellular and molecular responses of bovine milk phagocytes to persistent and transient strains of *Streptococcus uberis* causing mastitis.**

**Reviewer 3 comments:**

1. I agree with the authors that fluorescence observed in the unstimulated group could be due to autofluorescence. Yet, they should state the excitation and emitting wave length in the methods section. Please also specify the wave length at which the MTT (figure 4) and H2DCF-DA (figure 3) readings were performed.

**Response** The Flow cytometry lasers have been added to the Methods section of Phagocytosis assay and ROS assay, respectively .

- Adding "... with red laser (638 nm). " Please review line 168.
- Adding "... with green laser (561 nm)." Please review line 176.

2. In the paragraph added, they should italicize *S. uberis*.

**Response** They have been italicized. Please check line 429, 433, and 434.

3. Replace all gene names in figure 6B by the official gene names that are properly used in 6A.

**Response 3** The authors replaced all gene names in Figure 6B with the official gene names as Figure 6A.
